# Boosting precision crop protection towards agriculture 5.0 *via* machine learning and emerging technologies: A contextual review

**DOI:** 10.3389/fpls.2023.1143326

**Published:** 2023-03-22

**Authors:** Gustavo A. Mesías-Ruiz, María Pérez-Ortiz, José Dorado, Ana I. de Castro, José M. Peña

**Affiliations:** ^1^ Institute of Agricultural Sciences (ICA), Spanish National Research Council (CSIC), Madrid, Spain; ^2^ Escuela Técnica Superior de Ingeniería Agronómica, Alimentaria y de Biosistemas (ETSIAAB), Universidad Politécnica de Madrid, Madrid, Spain; ^3^ Centre for Artificial Intelligence, University College London, London, United Kingdom; ^4^ Environment and Agronomy Department, National Institute for Agricultural and Food Research and Technology (INIA), Spanish National Research Council (CSIC), Madrid, Spain

**Keywords:** precision agriculture (PA), artificial intelligence (AI), deep learning, unmanned aerial vehicles (UAV), decision support system (DDS), robotics

## Abstract

Crop protection is a key activity for the sustainability and feasibility of agriculture in a current context of climate change, which is causing the destabilization of agricultural practices and an increase in the incidence of current or invasive pests, and a growing world population that requires guaranteeing the food supply chain and ensuring food security. In view of these events, this article provides a contextual review in six sections on the role of artificial intelligence (AI), machine learning (ML) and other emerging technologies to solve current and future challenges of crop protection. Over time, crop protection has progressed from a primitive agriculture 1.0 (Ag1.0) through various technological developments to reach a level of maturity closelyin line with Ag5.0 (section 1), which is characterized by successfully leveraging ML capacity and modern agricultural devices and machines that perceive, analyze and actuate following the main stages of precision crop protection (section 2). Section 3 presents a taxonomy of ML algorithms that support the development and implementation of precision crop protection, while section 4 analyses the scientific impact of ML on the basis of an extensive bibliometric study of >120 algorithms, outlining the most widely used ML and deep learning (DL) techniques currently applied in relevant case studies on the detection and control of crop diseases, weeds and plagues. Section 5 describes 39 emerging technologies in the fields of smart sensors and other advanced hardware devices, telecommunications, proximal and remote sensing, and AI-based robotics that will foreseeably lead the next generation of perception-based, decision-making and actuation systems for digitized, smart and real-time crop protection in a realistic Ag5.0. Finally, section 6 highlights the main conclusions and final remarks.

## 1 Linking crop protection to the technological evolution of agriculture

Crop protection involves a large number of critical farming activities with a decisive impact on the viability and sustainability of agriculture. Throughout history, humans have developed new methods and practices to protect their crops. From ancient times to about 1950, agriculture 1.0 employed a large workforce to manually control crop pests (i.e., plant diseases, weeds and other plagues, both vertebrate and invertebrate), which produced low yields but in sufficient quantity to feed the population. In the late 1950s, agriculture 2.0 began with the use of synthetic pesticides and specialized machines to control the common crop pests. At that stage, agriculture evolved towards the economic edge, aiming to produce more food at a cheaper price, i.e., towards a more industrialized agriculture. At the end of the 20th century, agriculture 3.0 emerged with the idea of using new technologies and data-driven modeling as essential tools to take decisions and manage cropping systems. This disruptive concept led to the origin of precision agriculture, in which telematics, global navigation satellite systems (GNSS), machinery guidance, and sensing devices aimed to optimize the crop protection tasks, to reduce costs and environmental impacts of pesticides, and to improve food quality. What followed was a further step in the integration of geo-spatial technologies, computer sciences and digitization into the agricultural process, where sensors, mobile telephony, embedded systems, cloud computing, internet of things (IoT) and big data were incorporated on board of autonomous machinery, smart sprayers and actuators to facilitate the application of the precision crop protection paradigm within the concept of agriculture 4.0 (Zhai et al., 2020). Continuing this evolution, Agriculture 5.0 (Ag5.0) will promote a new era of intelligent crop management with automatized decision making processes, unmanned operations and progressively less human intervention supported by the latest Artificial Intelligence (AI) systems, advanced robotics, and powerful Machine Learning (ML) algorithms (Saiz-Rubio and Rovira-Más, 2020).

Modern agriculture will face in the next decades two immense challenges never seen in previous generations. The first one is the impact of climate change in agricultural systems (Hoegh-Guldberg et al., 2019), which causes destabilization of farming practices (Mulla et al., 2020) and irregular crop seasons due to excessive heat and water scarcity in large productive areas (Piao et al., 2019); (Falkland and White, 2020), which inevitably leads to the emergence of new invasive pests or the increased severity of existing ones. The second one is to produce food for a growing human and animal population, while ensuring food security by using fewer agrochemicals and imposing strict controls at all stages of the agricultural supply chain (van Dijk et al., 2020). In view on this imminent future, Ag5.0 must offer creative solutions based on AI, ML algorithms and other technological innovations that continuously interact with the crop and its environment, which will require undoubtedly transdisciplinary studies and interdisciplinary collaborations, where precision crop protection becomes a key discipline in the Ag5.0 revolution by implementing new procedures and strategies to drastically reduce the use of agrochemicals in the control of diseases, weeds and plagues.

## 2 The stages of precision crop protection: Perception, analysis and actuation

**Figure 1:**
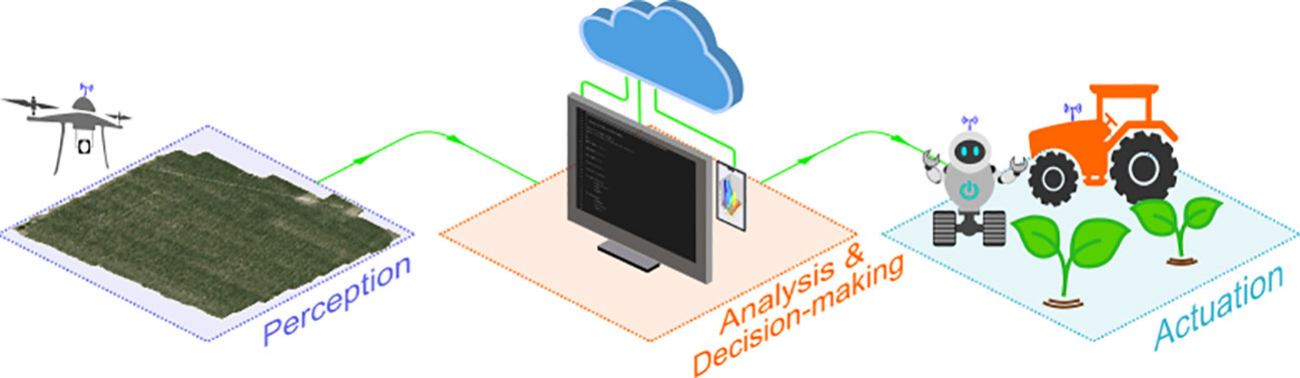
The main stages of precision crop protection.

The use of new technologies in crop protection aims at detecting and identifying the symptoms or problems caused by crop pests (Behmann et al., 2015), followed by a site-specific application of a chemical or mechanical control action. This process comprisesthe three main stages for pursuing a precision crop protection strategy, as follows (**Figure 1**): 1) perception, 2) analysis and, optionally (but recommendable) decision-making, and 3) actuation. The perception stage involves field inspection andacquisition of plant information (e.g., crop and/or weed imaging) through a sensor or camera mounted on an on-ground or a remotely-sensed platform, while the actuation stage consists on the application of a prescribed site-specific treatment with a smartequipment usually assisted by a GNSS receiver. The necessary link between perception and actuation is the analysis stage, which consists of in-depth evaluation of digital crop data by using diverse data-driven techniques and identifying targeting areas of crops with problems associated to diseases, weeds and plagues. The analysis stage also often includes the generation of management zones and treatment/prescription maps following a decision-making process, e.g. based on the outcomes of a decision support system (DSS).

Recent bibliographic reviews point out to Unmanned Aerial Vehicles (UAVs), innovative ML algorithms, and various robots and autonomous equipment as the most disruptive technology for each stage, respectively (Filho et al., 2020; Lima et al., 2020; Dainelli et al., 2021). UAVs are playing an important role in the perception stage due to their capability to capture crop data from large areas in a short time and with diverse types of cameras and sensors (e.g., RGB cameras, multi- and hyper- spectral sensors, thermal cameras, active sensors such as LiDAR, radar or sonar), which have led to significant progress in pest monitoring with the help of powerful analysis procedures, either by direct observation of the pest (e.g., weed patches), by diagnosis of the main symptoms of the disease (e.g., leave decay or thermal stress), or by detection of damages caused in the crop leaves and canopy (e.g., foliar losses due to a plague attack).

The analysis stage is the major challenge for many crops, probably being the bottleneck for the progress of precision crop protection. The ultimate objective of this stage is the accurate and timely detection of each crop-specific disease, weed or plague,whose complexity lies in the vast number of possible crop-pest scenarios with a diverse typology of associated symptoms, in addition to other environmental and cultural factors such as different weather conditions, soil properties, and farmers’ decisions on crop field management, which impact the type and degree of severity of pest occurrences (Oerke et al., 2012; Pätzold et al., 2020). This diversity of variables and factors can be addressed by ML methods with the ability to learn from experience (i.e., data) and integrate information from multiple sources. ML enables the analysis of massive amounts of crop and pest data over time by taking advantage of the continuous evolution of the hardware with increasingly powerful central (CPU), graphics (GPU) and tensor (TPU) processing units (Wang et al., 2019). As a result, ML can study the behavior of natural crop-pest systems by capturing and exploiting the underlying patterns in the data and build predictive/generative models accordingly for critical analytical tasks such as image classification, object detection, pattern recognition, geo-location, etc., aimed to propose solutions for complex crop protection challenges.

Finally, actuation is the task that leveraged large-scale viability of precision crop protection strategies, leading to great scientific and technological effort in the last decade to develop autonomous machinery, smart sprayers and agricultural robots that effectively implement site-specific crop management (Shafi et al., 2019; Lowenberg-DeBoer et al., 2021), either by direct treatment in real-time (Pérez-Ruiz et al., 2015) or, eventually, assisted by a prescription map (Fernández-Quintanilla et al., 2018) according to the principles established by the International Society of Precision Agriculture (ISPA, 2021).

## 3 ML taxonomy based on the tasks to be solved

**Figure 2:**
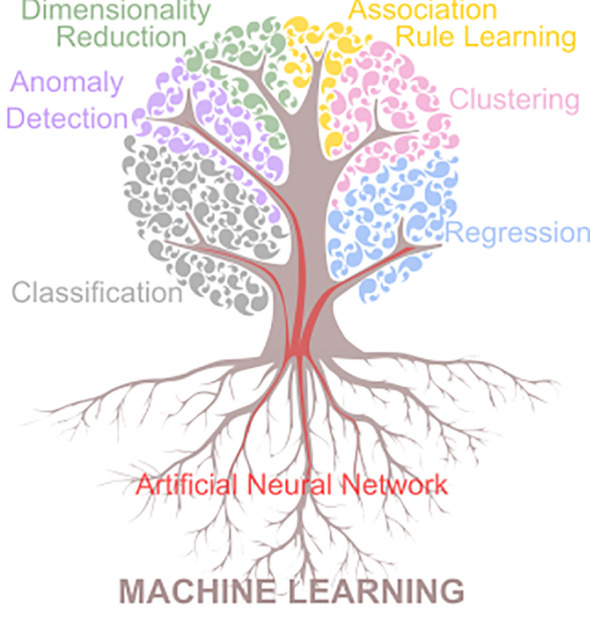
Taxonomy of machine learning according to the type of task to be solved.

The ML algorithms have been conventionally classified according to different criteria, based on: i) the nature of the model (full or partial probabilistic/generative model *vs*. discriminant model), ii) the type of reasoning applied (inductive or transductive, depending on whether the model performs a reasoning from observed training cases to general rules or the other way around, respectively), or iii) the data availability and the supervision process (unsupervised, supervised, semi-supervised and reinforcement learning). However, the extent of ML within the scope of precision crop protection is best described by an alternative criterion based on the task to be solved, which leads to an expanded taxonomy of six categories, as follows: classification, regression, clustering, anomaly detection, dimensionality reduction, and association rule learning. These six tasks can be addressed with traditional ML algorithms or, for some specific tasks mainly classification and regression, with the more advanced artificial neural network (ANN) models, which in turn also include Deep Learning (DL) algorithms (**Figure 2**).

### 3.1 Traditional ML algorithms

Traditional ML algorithms usually approach learning tasks by analyzing and interpreting input data with well-established architectures optimized for common computing resources, thus often achieving satisfactory results but with less accuracy and versatility than sophisticated ANN algorithms. Of the tasks listed above, classification is the most common in many disciplines, with well-known algorithms such as support vector machine (SVM), decision trees (DT), random forest (RF), K-Nearest Neighbor (k-NN), etc. Classification algorithms are part of the supervised learning type, aiming to categorize a certain set of structured or unstructured data in classes, being a binary classification when the objective is to predict the state of true or false, and a multi-category classification when there are more than two objective classes (Sen et al., 2020; Djafri and Gafour, 2022). These algorithms are used for predictive tasks in the fields of image analysis, video, object recognition, data mining, etc. (Kowsari et al., 2019), all of which are relevant to deal with the challenge of automatic identification, detection or classification of plant diseases, weeds and plagues. This objective also usually requires previous phases such as image/video preprocessing, segmentation and feature extraction that imply the use of other algorithms of the regression, clustering and dimensionality reduction typology.

The regression algorithms are also part of the supervised learning type and consist in relating continuous input and output variables through a function, which can be set by parametric or non-parametric approaches. In the former case, the output values are predicted by an explicit analytical formula that adjusts the known points by establishing and minimizing a cost function (e.g. linear regression) that link the input and output variables (Wei et al., 2015; Gaitán, 2020). In the latter case, a kernel function is defined to determine the prediction for the output based on similar experiences of the inputs, hence it depends on the correlation between the output and the known points surrounding the input (Čížek and Sadikoğlu, 2020). A form of regression that allows correction of overfitting is the regularization algorithms, which avoid generating low error (i.e., high accuracy) in the training but high error during the testing (Zou and Hastie, 2005). Common algorithms in this group are LASSO Regularization, Ridge Regularization and Elastic Net Regularization.

Within the two previous categories, the ensemble algorithms are the combination of predictions from various ML techniques applied to a single model improving predictive performance (Sagi and Rokach, 2018). In classification, an ensemble of classifiers is generally more accurate than the individual classifiers that compose it. Individual decisions are combined by weighted or unweighted votes in the classification of new examples (Hooftman et al., 2022), which allows a good balance between performance and computational cost (Telikani et al., 2022). The ensemble algorithms in regression improve accuracy while reduce bias and variance errors, avoiding over-adjustment when results deserve extra training (Ren et al., 2016). Some outstanding algorithms in this group are adaBoost, bootstrap aggregation (Bagging), category boosting (CatBoost), extremely randomized trees, gradient boosting machines (GBM), RF, stacked generalization (Stacking).

The clustering algorithms are part of the unsupervised and semi-supervised learning methods, which allow grouping the data into sets of similar objects to maximize the intra-cluster similarity and minimize inter-cluster similarity (Ezugwu et al., 2022). The partitional clustering applies techniques to obtain a single partition by an objective function of the input data, in a fixed number of clusters, using iterative relocation clusters and resulting in the best configuration of the total number of executions (Nanda and Panda, 2014). The hierarchical clustering performs the division of data (root node) by a sequence of nested partitions, known as tree type structures (dendrograms). This approach follows a type of pattern agglomerated (from bottom to top) or by divisive clustering (from top to bottom), with no need to define the number of clusters in advance (Murtagh and Contreras, 2012).

The dimensionality reduction algorithms transform a high-dimensional data set into a representative lower-dimensional subset, as not all data features may be equally relevant for the problem at hand, greatly reducing computational complexity (Xu et al., 2019). This technique is widely used for data preprocessing, by two different ways: i) feature selection, in which the input features are combined to obtain a new dataset with a smaller number of new variables that retain the original information based on the input components and projection, and ii) feature extraction, in which the most relevant features of the original dataset are kept by removing those features that contribute little or nothing to the output features (Chhikara et al., 2020).

The anomaly detection algorithms try to find patterns, outliers or some kind of exception in the data that do not conform to the expected behavior (Chandola et al., 2009), by mean of a function that decide about the detection of an unknown or heterogeneous novelty present in the datasets with a class imbalance (Guansong et al., 2022). Isolation Forest, One-Class SVM, and PCA-Based Anomaly Detection are the most common algorithms to detect anomalies with application in crop protection.

The association rule learning algorithms serve to find regularities present in parts of the dataset (descriptive rules) and generalize the dataset to enable predictions on new data (predictive rules) (Fürnkranz and Kliegr, 2015). These algorithms can identify an association rule in the form *A→B*, based on the indicators support, confidence and lift. Support from *A→B* is the percentage of all items in *A* and *B*. Confidence is the percentage of *A* and *B* by the percentage of *A*. Lift indicates the probability of *B* occurring since *A* has occurred (Hashimoto et al., 2018). Within this category, the algorithms Apriori and Eclat are the most popular.

### 3.2 Artificial neural networks and deep learning models

The ANN algorithms are highly customizable and flexible computing models roughly inspired by biological neural networks, based on creating connected networks of simple processing units (neurons) that together can learn complex patterns and solve undefined problems. The ANNs works as universal approximators for any mathematical function, whose learning process is based on training from large datasets through sequential computations until accurate patterns are obtained. Then, when new patterns are presented, ANNs are able to predict them. These algorithms are mainly applied in tasks of classification and regression, e.g. in approximation functions (i.e. mapping multiple inputs to a single output), pattern classification (i.e. identification of new patterns through association and pattern recognition), associative memories (i.e. pattern recognition from limited information in the subset of data), and generation of new significant patterns, which can help in the reconstruction of patterns with greater characteristics (Schmidhuber, 2015).

Neural networks with two or more layers are the conceptual basis to generate DL models, whose progress has been spectacular in recent years in all disciplines, even in precision crop protection (Ferentinos, 2018; Kamilaris and Prenafeta-Boldú, 2018; Xia et al., 2018; Farooq et al., 2019; Hasan et al., 2021; Rakhmatulin et al., 2021; Allmendinger et al., 2022; Tugrul et al., 2022; Rai et al., 2023). DL algorithms transform data to construct complex concepts in a hierarchical structure with several levels of abstraction, so that the higher levels are composed of the characteristics of the lower levels (LeCun et al., 2015). The great potential of DL in many fields employing image analysis is allowing small data sets to be fitted to pre-trained models with different data, reducing training time and optimizing hardware resources (Kamilaris and Prenafeta-Boldú, 2018). DL covers different approaches suited to specific problems, for example, convolutional neural networks (CNNs) are used in computer vision and image classification, recurrent neural networks (RNNs) are used for prediction and language modelling, autoencoder is used in dimensionality reduction, and generative adversarial networks (GANs) are used in the generation of new images (Sarker, 2021).

CNN architectures for image classification is the most common application of DL in precision crop protection. The CNN algorithms find the features of objects of interest by self-learning from the image data, in contrast to traditional ML algorithms that require the user to establish such features (Hong et al., 2020). Performance of CNNs varies depending of number of parameters and convolutional layers (network depth), which in turn is directly constrained by the power of the available computing resources (**Table 1**). A broader application of CNN-based classifiers is object detection, which overcomes the issue of visual recognition in multi-class domains and object labelling in computer vision. Examples of CNN architectures for object detection and classification implemented in crop protection include Region-based Convolutional Neural Network (R-CNN) (Girshick et al., 2014), Fast R-CNN (Girshick, 2015), Faster R-CNN (Ren et al., 2015), You Only Look Once (YOLO) (Redmon et al., 2016), Single Shot Detector (SSD) (Liu et al., 2016), Feature Pyramid Networks (FPN) (Lin et al., 2017a), RetinaNet (Lin et al., 2017b) and Mask R-CNN (He et al., 2017).

**Table 1:** Characteristics of the deep learning architectures most commonly used in crop protection.

CNN Architecture	Depth (layers)	Million parameters	Top-5 Accuracy % *
LeNet-5 (LeCun et al., 1998)	5	0,06	–
AlexNet (Krizhevsky et al., 2012)	8	60	84.6
VGG-Net (Simonyan and Zisserman, 2014)	16	138.4	90.1
GoogLeNet (Szegedy et al., 2015)	22	4	92.2
ResNet (He et al., 2016)	152	60.4	93.1
Xception (Chollet, 2016)	126	22.8	94.5
DenseNet (Huang et al., 2016)	402	20.2	93.6
MobileNet (Howard et al., 2017)	55	4.3	89.5

*ImageNet validation dataset.

## 4 Scientific impact and relevant contributions of ML in precision crop protection

**Figure 3:**
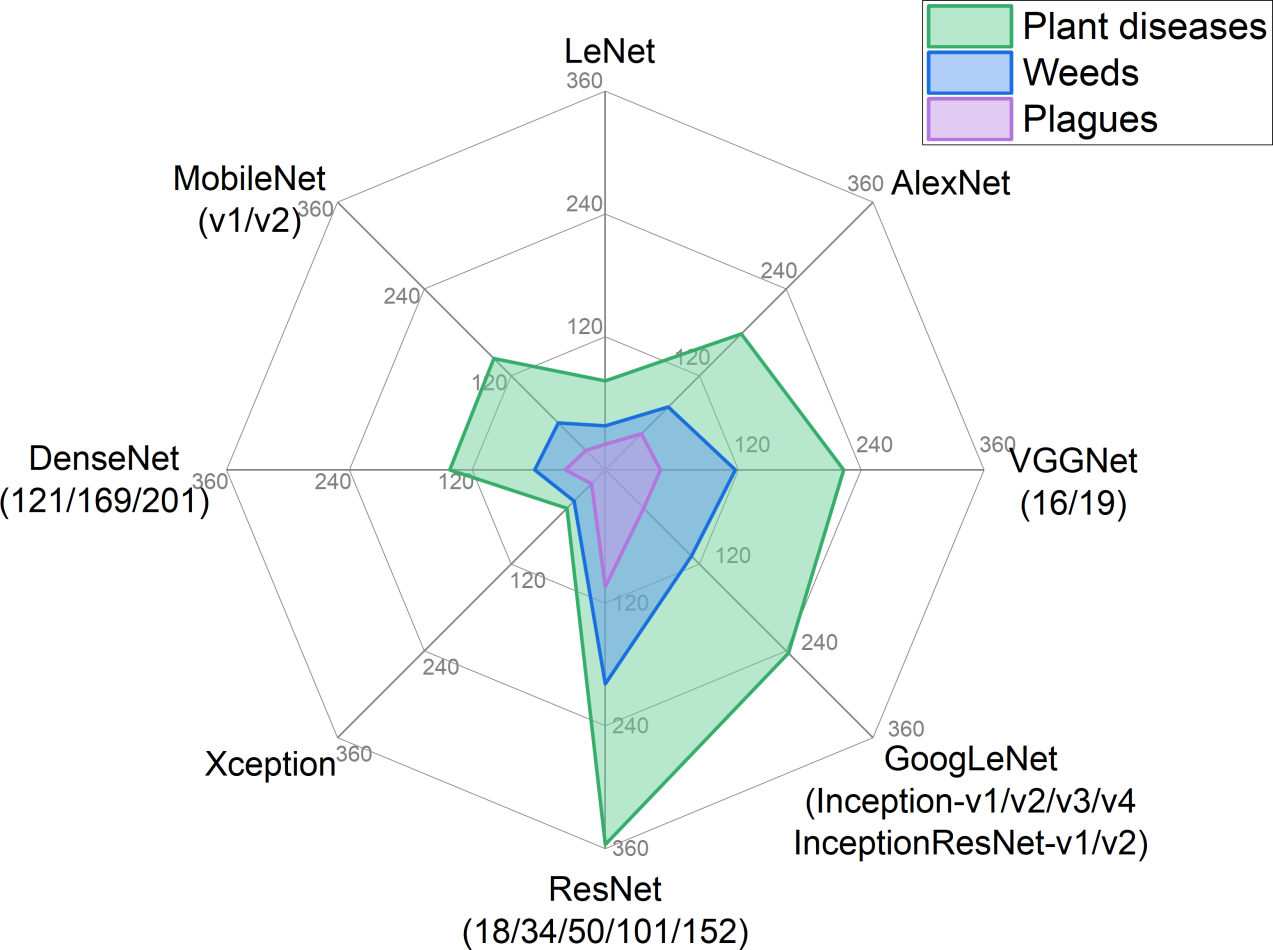
Number of publications of CNN architectures commonly used in the three domains of precision crop protection (crop diseases, weeds and crop plagues) from 2010 to 2022 (source: Scopus). Figure compiled with the conjunction of “CNN architecture” and each of the three crop protection domains (crop diseases, weeds and crop plagues) as search criteria within the article title, abstract and keywords.

An extensive bibliometric study of the Scopus database (www.scopus.com) revealed 107 traditional ML algorithms and 18 ANN models applied in all disciplines between 2010 and 2022, of which 105 and 17 algorithms, respectively, have been implemented in precision crop protection objectives with diverse degree of contribution in the domains of crop diseases, weeds and plagues (**Table 2**). SVM topped the list of traditional algorithms applied in precision crop protection objectives with >1,700 publications, followed by linear regression (LR) and Stacking with >1,500 publications each one. Principal Component Analysis (PCA), RF and DT are other algorithms with high impact reaching more than 1,100 publications each. A four group of relevant algorithmsis formed by Bagging, logistic regression (LoR), k-NN and k-means clustering, which appear in more than 500 publications of precision crop protection. Some algorithms rank relatively high in terms of their use in precision crop protection in comparison to all disciplines (PCP/All), such as k-NN, simple linear iterative clustering (SLIC), stacking and stepwise discriminant analysis (SDA) (>10% PCP/All), or in comparison to precision agriculture (PCP/PA), such as Gaussian Mixture Regression (GMR) (>70% PCP/PA). Among the ANN models, convolutional neural networks (CNNs) are by far the most widely used in precision crop protection with >1,200 publications, mainly focused on detecting and classifying crop diseases, weeds or plagues with image-based technology, with ResNet, GoogLeNet and VGGNet being the most applied models, and to a lesser extent LeNet and Xception models (**Figure 3**).

**Table 2:** Numbers publications of machine learning algorithms according to the proposal taxonomy (source Scopus).

							Number of ML Publications				
Algorithm	Task to be solved (†)						In Precision Crop Protection (PCP)				PCP/PA (‡)
	Clas	Regr	Clus	Anom	Dim	Asso	Diseases	Weeds	Plagues	In PA	
Traditional:											
Support Vector Machine (SVM)	✓	✓					612	560	540	>1,000	***
Linear Regression (LR)		✓					287	699	693	>10,000	***
Stacked Generalization (Stacking)	✓	✓					292	393	812	>1,000	***
Principal Component Analysis (PCA)	✓				✓		383	458	518	>10,000	***
Random Forest (RF)	✓	✓					374	437	395	>1000	***
Decision Trees (DT)	✓						311	380	414	>1000	***
Bootstrap Aggregation (Bagging)	✓	✓					195	356	414	>1000	***
Logistic Regression (LoR)	✓						129	171	451	>1000	***
k-Nearest Neighbors (k-NN)	✓						276	195	247	>1000	***
K-Means Clustering			✓				210	185	152	>1000	***
Hierarchical Clustering			✓				114	143	182	>1000	***
Linear Discriminant Analysis (LDA)	✓						158	125	129	>1000	***
Naïve Bayes	✓						146	95	169	>1000	****
Regression Trees		✓					94	134	124	>1000	***
Factor Analysis					✓		37	80	208	>1000	***
Stochastic Gradient Descent	✓						117	84	74	>100	****
Partial Least Squares Regression (PLSR)		✓					101	127	40	>1000	***
Support Vector Regression (SVR)		✓					75	75	84	>1000	***
Expectation Maximization			✓				45	40	131	>1000	***
Singular Value Decomposition (SVD)					✓		24	37	151	>1000	***
LASSO		✓					39	44	118	>100	***
AutoEncoder					✓		53	46	102	>100	****
Multi Dimensional Scaling (MDS)					✓		58	68	70	>1000	***
Self-Organizing Maps	✓						57	63	71	>1000	***
Extreme Learning Machine (ELM)		✓					59	64	64	>1000	***
Gaussian Mixture Model (GMM)			✓				49	46	91	>100	****
AdaBoost	✓	✓					58	46	74	>100	***
Fuzzy c-Means (FCM)			✓				61	59	46	>100	***
Partial Least Squares Discriminant Analysis					✓		63	53	19	>1000	**
Fuzzy Clustering			✓				33	56	43	>100	***
Independent Component Analysis (ICA)					✓		22	22	85	>100	****
Ridge Regression (RR)		✓					15	25	77	>100	***
Extreme Gradient Boosting (xGBoost)	✓	✓					24	25	64	>1000	***
Stepwise Regression		✓					24	40	39	>1000	**
Quadratic discriminant analysis	✓						51	26	25	>100	****
Gaussian Process Regression (GPR)		✓					31	19	40	>100	***
Polynomial Regression		✓					15	33	41	>100	***
Principal Component Regression (PCR)		✓					33	31	22	>100	***
Boosted Trees (BoT)	✓	✓					22	20	25	>100	***
Simple Linear Iterative Clustering (SLIC)			✓				31	30	4	>100	****
Apriori						✓	8	14	42	>100	***
Subset Selection					✓		18	21	23	>100	***
Quantile Regression		✓					6	15	39	>100	***
Ordinary Least Squares (OLS) Regression		✓					6	13	41	>100	***
DBSCAN			✓				16	22	17	>100	***
Model Trees		✓					13	19	21	>100	**
Spectral Clustering			✓				5	12	35	>100	***
Gradient Boosting Machines (GBM)	✓	✓					10	9	28	>100	***
Poisson Regression		✓					2	14	30	>100	***
Multivariate Adaptive Regression Splines (MARS)		✓					10	16	20	>100	**
Minimum Spanning Trees			✓				3	11	27	>100	***
t-Distributed Stochastic Neighbor Embedding (t-SNE)					✓		13	6	21	>100	***
Stepwise Multiple Linear Regression (SMLR)		✓					17	16	5	>100	***
Stepwise Discriminant Analysis (SDA)							20	13	4	>1,000	****
Generalized Regression Neural Network (GRNN)		✓					12	12	12	>100	***
Maximum likelihood classifier (MLC)	✓						7	24	3	>100	**
One Rule	✓						6	6	21	>100	***
Kernel Principal Component Analysis (k-PCA)					✓		10	6	13	>10	****
One Class SVM				✓			8	5	16	>10	****
Gradient Boosted Regression Trees	✓	✓					11	11	4	>100	***
Quality Threshold			✓				6	7	12	>100	***
Gaussian Naive Bayes	✓						10	7	7	>10	****
Fisher’s linear discriminant analysis	✓						10	4	9	>10	****
Fuzzy K-Means			✓				4	14	5	>100	***
Bagging Trees (BaT)	✓	✓					10	6	7	>100	***
Multiple-Kernel Learning (MKL)	✓						2	6	14	>10	****
Isomap					✓		5	1	15	>100	***
Kernel Ridge Regression (KRR)		✓					3	5	13	>10	***
Extremely Randomized Trees	✓	✓					6	7	7	>10	***
Rotation Forest	✓	✓					7	7	3	>100	***
Isolation Forest				✓			5	2	10	>10	****
Multinomial Naive Bayes	✓						2	2	13	>10	****
Laplacian Eigenmaps					✓		2	1	13	>10	***
Elastic Net Regression		✓					2	3	10	>10	***
LASSO Regularization		✓					1	3	11	>10	****
K-Medoids Clustering			✓				1	3	9	>10	***
Least-Angle Regression (LAR)		✓					2	3	7	>10	***
Mean Shift Clustering			✓				1	7	4	>10	****
Locally Weighted Regression (LWR)		✓					1	2	9	>100	**
FP-growth						✓	2	3	6	>10	****
Elastic Net Regularization		✓					1	2	8	>10	***
Zero-Shot Learning							3	2	6	>10	****
Locality Preserving Projections					✓		3	1	6	>10	***
Bayesian Network Classifier	✓						2	4	4	>10	****
Forward Feature Selection					✓		4	3	2	>10	***
Voting Classifier	✓	✓					2	3	3	>10	****
Decision Stump	✓						1	4	3	>10	***
Local Linear Embedding (LLE)					✓		5	1	2	>10	***
Ordinal Regression		✓					–	1	6	>10	**
Local Outlier Factor (LOF)				✓			–	–	6	>10	***
Gaussian Mixture Regression (GMR)		✓					–	–	6	>1	*****
Random Subspace Methods	✓	✓					1	2	2	>10	***
Category Boosting (CatBoost)	✓	✓					–	–	5	>10	**
Clustering Large Applications (CLARA)			✓				2	1	2	>10	***
DENCLUE			✓				2	1	2	>10	****
Ridge Regularization		✓					–	2	2	>10	***
Bayesian Linear Regression		✓					1	1	2	>10	**
Sammon Mapping					✓		1	1	2	>10	***
Eclat						✓	1	1	1	>10	***
Relevance Vector Regression		✓					–	–	2	>10	***
Bernoulli Naive Bayes	✓						–	–	2	>10	***
K-Modes Clustering			✓				–	–	2	>1	****
Regularized Linear Discriminant Analysis (RLDA)					✓		–	2	–	>10	***
Zero Rule	✓						–	–	1	>1	***
Gradient Descent Regression		✓					–	–	1	>1	****
Fast-MCD				✓			–	–	–	>1	–
PCA-Based Anomaly Detection				✓			–	–	–	>1	–
Artificial Neural Networks:											
Convolutional Neural Network (CNN)	✓	✓					528	395	339	>1,000	****
Back Propagation	✓	✓					190	176	189	>1,000	***
Radial Basis Function (RBF)	✓						149	135	167	>1,000	***
Recurrent Neural Network (RNN)	✓	✓					92	80	159	>1,000	***
Multi-Layer Perceptron (MLP)	✓	✓					65	66	83	>1,000	***
Generative Adversarial Network (GAN)		✓					86	43	85	>100	****
Deep Belief Network (DBN)	✓	✓					46	35	45	>100	****
Probabilistic Neural Network (PNN)	✓						52	22	21	>100	****
Boltzmann Machine		✓					24	15	36	>100	****
Restricted Boltzmann Machine (RBM)		✓					18	9	29	>100	****
Stacked Autoencoder	✓			✓			8	12	13	>100	****
Learning Vector Quantization (LVQ)	✓						14	8	3	>100	***
Kohonen’s Self-Organizing Map (SOM)	✓						4	4	5	>10	***
Single-Layer Perceptron (SLP)	✓	✓					4	4	6	>10	***
Hopfield Networks		✓					3	2	8	>10	****
Bayesian Regularized Neural Networks	✓						–	–	4	>10	**
Supervised Kohonen Network (SKN)	✓						6	9	–	>10	*****
Counter-Propagation ANNs (CP-ANNs)	✓						–	–	–	>1	–

(†) Clas, Classification; Regr, Regression; Clus, Clustering; Anom, Anomaly Detection; Dim, Dimensionality Reduction; Asso, Association Rule Learning.

(‡) ***** >50%; **** >25%; *** >10%; ** >5%; –No cases. ✓ Indicates that this algorithm was used in the task to be solved.

**Figure 4:**
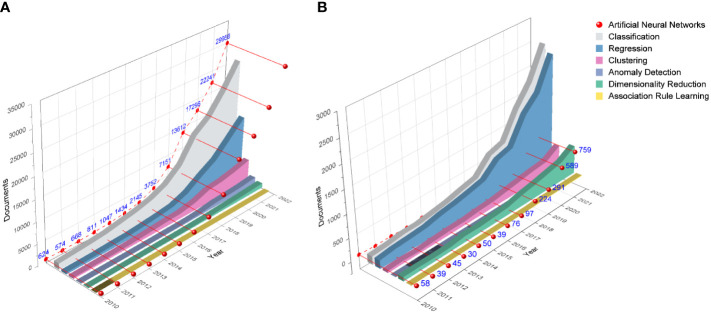
Publications trends (2010 – 2022) of traditional ML algorithms (colored solid areas) and ANNs (dashed red line) in all disciplines **(A)**, and for precision crop protection applications **(B)**, according to the proposed taxonomy (source: Scopus).

A temporal analysis on ML-based publications shows that the adoption of ML algorithms has increased steadily year on year across all disciplines over the last decade (**Figure 4A**), which in turn is boosting the development of precision crop protection strategies (**Figure 4B**). Comparing the trends in both figures, peak values were reached in the last year in all cases, with classification and regression tasks being the most common by far in the group of traditional ML algorithms (55% and 29% across all cases and 47% and 41% in precision crop protection, respectively), followed by clustering, anomaly detection and dimensionality reduction tasks in the case of all disciplines, with considerably less impact (11%, 3% and 2%, respectively), and a negligible value for association rule learning. However, the dimensionality reduction algorithms were much more widely used in precision crop protection (11%) than the other three categories. In the case of ANN algorithms, their use has increased significantly in the last five years, counting 29,956 (**Figure 4A**) and 759 new publications (**Figure 4B**) in 2022 across all disciplines and in precision crop protection, respectively. Compared to the traditional ML algorithms, ANN algorithms remain at the highest rates since 2018 across all disciplines, but still do not exceed traditional classification algorithms in precision crop protection, although they did overcome dimensionality reduction algorithms in 2019 and regression algorithms in 2022.

These positive indicators on the growing impact of ML in precision crop protection are supported by numerous applications and case studies outlined in detail in quite a few recent scientific reviews (Behmann et al., 2015; Liakos et al., 2018; Muppala and Guruviah, 2020; Chadha et al., 2021; Saleem et al., 2021). An in-depth analysis of some relevant publications reveals key challenges addressed by diverse image-based or sensor technology together with ML algorithms in the specific domains of crop diseases (**Table 3**), weeds (**Table 4**) and plagues (**Table 5**), as discussed hereunder.

**Table 3:** Relevant investigations on ML algorithms in the domain of crop diseases.

Image/sensor technology	Crop/Pathogen type	Main objective	Task to be solved	ML Algorithm	Reference
Field spectroradiometer	Wheat/fungal	Detection and monitoring of powdery mildew (*Erysiphe graminis*)	Regression, ensemble	PLSR, SVM, RF	(Feng et al., 2022)
	Potato/fungal	Pre- and post-symptomatic detection of late blight (*Phytophthora infestans*) in leaves	Classification, ensemble	RF, PLS-DA	(Gold et al., 2020)
	Avocado/fungal, nutrient deficiency	Early and late detection of laurel wilt (*Raffaelea lauricola*), N deficiency and Fe deficiency in leaves	Classification	DT, MLP	(Abdulridha et al., 2018)
	Tomato/bacterial, fungal	Discrimination of bacterial spots (*Xanthomonas vesicatoria*) among others fungal diseases (e.g. Late blight and target) with similar symptoms	Dimensionality reduction, classification	PCA,k-NN	(Lu et al., 2018)
	Strawberry/fungal	Asymptomatic and symptomatic detection of anthracnose crown rot (*Colletotrichum*)	Classification, regression	FDA, SDA, k-NN	(Lu et al., 2017)
	Avocado/fungal	Early and late detection of laurel wilt (*Raffaelea lauricola*) & phytophthora root rot	Classification	MLP, RBF	(De Castro et al., 2015)
On-ground hyperspectral camera	X Sugar beet/fungal	Early detection of rhizoctonia root and crown rot (*Rhizoctonia solani*) in leaves	Classification, regression, ensemble	PLS, RF, k-NN, Linear SVM, Radial SVM	(Barreto et al., 2020)
	Seed potatoes/viral	Real-time detection of potato virus y (*pvy, genus potyvirus*, family *potyviridae*) in tractor-mounted imagery	Classification	Fully CNN	(Polder et al., 2019)
	Wheat/fungal	Early detection of head blight (*Fusarium*)	Classification	VGG, RNN	(Jin et al., 2018)
	Tobacco/viral	Early (pre-symptomatic) detection of tobacco mosaic virus (tmv) in tobacco leaves	Classification, regression, ensemble	PLS-DA, RF, SVM, BPNN, ELM, LS-SVM	(Zhu et al., 2017)
Satellite multi-spectral and thermal images	Coffee/bacterial	Detection and progress of bacterial blight (*Pseudomonas syringae pv. Garcae*)	Classification, ensemble	RF, SVM, Naïve Bayes	(de Carvalho Alves et al., 2022)
Airborne hyperspectral and thermal images	Olive and almond trees/bacterial, fungal	Detection of *Xylella fastidiosa* (bacteria) and *Verticillium dahlia* (fungus) symptoms across species and pathogens	Classification, clustering	SVM, RF	(Zarco-Tejada et al., 2021)
	Olive trees/bacterial	Previsual symptoms detection of *Xylella fastidiosa* infection	Classification, ensemble	LDA, SVM, RBF, neural network ensemble	(Zarco-Tejada et al., 2018)
	Olive trees/fungal	Early detection and quantification of Verticillium wilt (*Verticillium dahlia*)	Classification	LDA, SVM	(Calderón et al., 2015)
Airborne hyperspectral & UAV-based multispectral images	Citrus trees/bacterial	Identification of Huanglongbing (HLB) with two aerial imaging systems	Regression, Classification	Stepwise regression, SVM, LDA, QDA	(Garcia-Ruiz et al., 2013)
UAV-based hyperspectral images	Wheat/fungal	Detection of yellow rust (*Puccinia striiformis* f. Sp. Tritici (pst)) across crop cycle	Classification, regression	ResNet, RF	(Zhang et al., 2019a)
UAV-based multispectral images	Apple trees/bacterial	Detection of apple fire blight (*Erwinia amylovora*)	Dimensionality reduction, anomaly detection, classification	mRMR, Isolation forest, DT, RF, SVM	(Xiao et al., 2022)
	Banana/bacterial, viral	Discrimination between Banana Xanthomonas wilt (BXW) and Bunchy top virus (BBTV) diseases	Classification, dimensionality reduction	VGG16, ResNet50	(Selvaraj et al., 2020)
	Pear trees/bacterial	Detection of fire blight (*Erwinia amylovora*)	Classification	SVM, RBF	(Bagheri, 2020)
Repository of RGB images of leaves	Grapes/fungal	Diagnosing *black rot*, *black measles* (esca) and *leaf blight* diseases in leaves for potential use in mobile devices	Classification	AlexNet, MobileNet, ShuffleNet	(Tang et al., 2020)
	Corn/fungal	Real-time detection of common rust and northern leaf blight damages in leaves	Classification	CNN	(Mishra et al., 2020)
	Tomato/bacterial, fungal, viral	Real-time detection of tomato mosaic virus in leaves	Classification	AlexNet, SqueezeNet	(Durmuş et al., 2017)
On-ground RGNIr for leaves	Pear trees/bacterial	Detection of fire blight *(Erwinia amylovora)*	Classification	SVM, RBF	(Bagheri, 2020)

**Table 4:** Relevant investigations on ML algorithms in the domain of crop weeds.

Image/sensor technology	Crop/Weed species	Main objective	Task to be solved	ML Algorithm	Reference	
Field spectroradiometer	No crop/*Sorghum halepense*	Differentiating glyphosate- resistant and susceptible Johnsongrass plants	Classification, regression, ensemble	k-NN, RF, SVM with FLDA	(Huang et al., 2022)
	No crop/*Amaranthus species*	Spectral discrimination of six Amaranthus species	Classification	SVM, Generalized Linear Model, DT, Naïve Bayes	(Sohn et al., 2021)	
	No crop/*Cyperaceae* weeds	Spectral discrimination of *Cyperus esculentus* clones and morphologically similar weeds	Classification, dimensionality reduction	RF, regularized LoR, PLS-DA	(Lauwers et al., 2020)	
	Wheat, broad bean/Cruciferous weeds	Selecting optimal spectral bands for image-based weed detection	Classification	MLP, RBF	(de Castro et al., 2012)	
	Wheat/*Avena sterilis*, *Phalaris* spp.	Selecting suitable timeframe and spectral regions for discriminating wheat and two grass weeds	Classification, Dimensionality reduction	Stepwise discriminant analysis	(Gómez-Casero et al., 2010)	
On-ground hyperspectral camera	Spring wheat, barley/*Kochia scoparia*	Differentiating glyphosate- and dicamba- resistant and susceptible Kochia plants	Classification	SVM with RBF kernel	(Nugent et al., 2018)	
	No crop/*Amaranthus palmeri*	Differentiating glyphosate- resistant and susceptible Palmer amaranth plants	Classification, dimensionality reduction	MLC, FLDA	(Reddy et al., 2014)	
	Rice/*Echinochloa crusgalli*, *Oryza sativa*	Discrimination of two weed species (Barnyard grass and weedy rice) with similar spectral signatures	Classification, regression, ensemble	RF, SVM, feature selection: successive projection algorithm (SPA).	(Zhang et al., 2019b)	
	Maize/*Convolvulus arvensis*, *Rumex*, *Cirsium arvense*	Discrimination of three weed species	Classification, dimensionality reduction, ensemble	k-NN, RF, PCA	(Gao et al., 2018)	
Satellite multi-spectral images	Winter wheat/Cruciferous weeds	Mapping cruciferous weed patches in multiple fields at broad scale	Classification	MLC	(de Castro et al., 2013)	
UAV-based multi-spectral and/or RGB images	Wheat/blackgrass weed	Spectral analysis and mapping of blackgrass weed	Classification, dimensionality reduction	Feature selection, RF with Bayesian optimization	(Su et al., 2022)	
	Sunflower, cotton/broad-leaved & grass weeds	Discrimination between broad-leaved and grass weeds	Classification	ANN-based MLP	(Torres-Sánchez et al., 2021)	
	Vineyard/*Cynodon dactylon*	Detection of bermudagrass in complex scenarios with cover crop, bare soil and vines	Classification	DT	(de Castro et al., 2020)	
	Sunflower, cotton/Several weeds	Early-season weed mapping between and within crop rows	Classification, ensemble	RF	(De Castro et al., 2018)	
	Sunflower, maize/Several weeds	Selecting patterns and features for between and within crop-row weed mapping	Classification, clustering	K-means clustering, SVM	(Pérez-Ortiz et al., 2016)	
	Sunflower/Several weeds	Comparing several ML paradigms to distinguish both weeds outside and within crop rows	Classification, clustering	k-means clustering, Linear SVM-based approximation, k-NN, SVM	(Pérez-Ortiz et al., 2015)	
On-ground RGB imagery	Tomato/Several weeds	Object detection and classification of five weed species	Classification	RetinaNet, Faster RCNN, YOLOv7	López-Correa et al., 2022)	
	Potato/*Chenopodium album*	Comparing CNN-based method to detect Chenopodium album in the crop field	Classification	GoogLeNet, VGG-16, EfficientNet	(Hussain et al., 2021)	

**Table 5:** Relevant investigations on ML algorithms in the domain of crop plagues.

Image/sensor technology	Crop/Plague type	Main objective	Task to be solved	ML Algorithm	Reference
VNIR-SWIR spectroradiometer	Cotton/Worm	Modeling the spectral response of cotton plants under the *Fall armyworm* attacks	Classification	RF, DT, MLP, XGBoost, SVM, Naïve Bayes, LoR, k-NN	(Ramos et al., 2022)
Portable NIR spectroscopy & e-nose sensors	Wheat/Aphid	Detecting level of *Oat aphids* infestation and predicting insect number	Classification, regression	ANN-based regression models, Bayesian Regularization, SVM	(Fuentes et al., 2021)
UAV-based multispectral imagery	Cotton/Spider mite	Detection of two-spotted spider mite in crop fields	Classification	SVM, AlexNet	(Huang et al., 2018)
RGB imagery from traps	No crop/Pest moth	Detecting *Helicoverpa assulta*, *Spodoptera litura* and *Spodoptera exigua* in pheromone trap images	Classification	Faster-RCNN ResNet, Faster RCNN Inception, R-FCN ResNet, RetinaNet ResNet, RetinaNet Mobile, SSD Inception	(Hong et al., 2020)
	No crop/Multi-class plagues	Detection and classification of multi-class plague species in trap images	Classification	VGG16, ZF, ResNet50, ResNet101	(Liu et al., 2019)
Repository of insect images	No crop/Multi-class plagues	Detection and classification of multi-class plague species in insect images	Classification	VGG19, SSD, Fast RCNN	(Xia et al., 2018)
On-ground RGB imagery	Tomato and pepper/Pest	Vision-based automated detection and identification of *Bemisia tabaci & Trialeurodes vaporariorum*	Classification	k-NN, MLP, SSD, Faster-RCNN	(Gutierrez et al., 2019)
	Strawberry/Thrips	Real-time detection of thrips (Thysanoptera) in flower images	Classification	SVM	(Ebrahimi et al., 2017)

In recent literature, one major goal is to study slight alterations in crop spectral information or other sensory components (e.g., odors or flavors) associated with pathogen infestations or with damages caused by a plague attack (**Tables 3**, **5**). This is generally done with on-ground measurements of plant leaves or canopies collected by hyperspectral cameras, field spectroradiometers or other portable sensors (e.g., e-nose sensor), and analyzing the spectral signatures or sensor data with ML classification and/or regression algorithms, aiming to discriminate between healthy and infested plants at the earliest possible stages or to model/predict the spectral response of infested plants. Dimensionality reduction algorithms (e.g., PCA, PLS-DA) is also often applied to transform large datasets into a lower dimensional space to facilitate further analysis. This approach was used at the disease domain, e.g., for early stage classification of anthracnose crown rot disease (by *Colletotrichum fungus*) in strawberry crop with SDA, FLDA and k-NN algorithms (Lu et al., 2017), classifying pre- and post- symptomatic fungal infestations of late blight (*Phytophthora infestans*) in potato leaves with PLS-DA and RF algorithms (Gold et al., 2020), monitoring the rate of fungal powdery mildew (*Erysiphe graminis*) disease in wheat with PLSR, SVR and RFR algorithms (Feng et al., 2022), and pre-symptomatic detection of tobacco mosaic virus in tobacco leaves with PLS-DA, RF, SVM, BPNN, ELM and LS-SVM (Zhu et al., 2017); while at the plague domain was used, e.g., for predicting and classifying oat aphids (*Rhophalosiphum padi*) number in wheat cultivation with ANNs models applied to NIR and e-nose data (Fuentes et al., 2021), and spectralmodelling of cotton plants against fall armyworm (*Spodoptera frugiperda*) attacks with RF, XGBoost, Naïve Bayes, LoR, SVM, MLP and k-NN algorithms (Ramos et al., 2022). These tools have also shown effective in other more complex scenarios dealing with hyperspectral discrimination of various diseases or other stresses/deficiencies that may cause similar symptomatology, such as fungal *Rhizoctonia* root and crown rot (*Rhizoctonia solani*) diseases in sugar beet leaves with PLS, RF, k-NN, and SVM (Barreto et al., 2020), bacterial spots (*Xanthomonas vesicatoria*) disease among other fungal diseases (late blight and target) in tomato leaves with PCA and k-NN algorithms (Lu et al., 2018), fungal laurel wilt (*Raffaelea lauricola*) and *Phytophthora* root rot diseases in avocado trees with ANN-based MLP and RBF models (De Castro et al., 2015), and laurel wilt disease against N and Fe nutrient deficiencies in avocado leaves with DT and MLP (Abdulridha et al., 2018).

At the domain of weed science (**Table 4**), field hyperspectral technology have been routinely tested to find the best spectral regions or vegetation indices to discriminate between weeds and crops at different phenological stages (Peña-Barragán et al., 2006; Basinger et al., 2020), generally with the aim of extrapolating results for remote sensing applications (Gómez-Casero et al., 2010; de Castro et al., 2012) in the context of site-specific weed management. Moreover, ML algorithms have recently dealt with challenging issues such as: 1) discrimination of multiple weed species with similar spectral response, such as Barnyard grass (*Echinochloa crusgalli*) and weedy rice (*Oryza sativa*) in rice crops with RF, SVM and SPA (Zhang et al., 2019b), *Convolvulus arvensis*, *Rumex*, and *Cirsium arvense* in maize crops with PCA, k-NN and RF (Gao et al., 2018), six *Amaranthus* species with SVM, DT and Naïve Bayes (Sohn et al., 2021) and *Cyperus esculentus* clones and morphologically similar weeds with RF, regularized LoR and PLS-DA (Lauwers et al., 2020), or 2) differentiation of herbicide- resistant and susceptible Palmer amaranth (*Amaranthus palmeri*) plants, Kochia (*Kochia scoparia*) plants or Johsongrass (*Sorghum halepense*) plants with MLC and FLDA (Reddy et al., 2014), SVM with RBF kernel (Nugent et al., 2018), and k-NN, RF and SVM with FLDA (Huang et al., 2022), respectively.

Disease, weed and plague detection and mapping with remote sensing have been particularly benefited from the adoption of ML algorithms (de Castro Megías et al., 2021; Lassalle, 2021; Roslim et al., 2021). In this context, proper selection of spectral and spatial image resolutions, as well as the optimal timing, is crucial to achieve satisfactory results (Peña et al., 2015; Khanal et al., 2017), which promotes the use of UAVs or manned aircrafts to the detriment of satellites in precision crop protection. Nonetheless, ML and satellite imagery can be useful in broad-scale applications, e.g., for evaluating integrated bacterial blight disease management in coffee plantations with several ecological variables (*Landsat-8* surface reflectance values and VIs, relief morphometry and hydrological attributes) by using RF, SVM and Naïve Bayes (de Carvalho Alves et al., 2022), or for mapping cruciferous weed patches in multiple winter wheat fields with *QuickBird* satellite imagery by using MLC (de Castro et al., 2013). Thermal and hyper-spectral aerial images with capability to capture slight variations in crop temperature and in narrow spectral bands associated to certain physiological indicators, respectively are commonly used in early detection of crop diseases, such as for identifying bacterial *Huanglongbing* (HLB) disease in citrus trees with stepwise regression, SVM, LDA and QDA (Garcia-Ruiz et al., 2013), fungal Verticillium wilt *(Verticillium dahlia)* disease in olive trees with LDA and SVM (Calderón et al., 2015), bacterial *Xylella fastidiosa* infections in olive trees (Zarco-Tejada et al., 2018), and fungal yellow rust (*Puccinia striiformis*) across crop cycle in wheat with RF and CNN-based Inception-ResNet blocks (Zhang et al., 2019a). SVM with a Gaussian kernel and RF algorithms also helped to diminish the uncertainty of distinguishing trees affected by diverse biotic (i.e., infections by *Xylella fastidiosa* and *Verticillium dahlia* pathogens) and abiotic (i.e., water status) stressors that produce analogous symptoms on spectral traits in olive and almond orchards (Zarco-Tejada et al., 2021).

Most recent research in precision crop protection relies on analyzing UAV images collected with low-cost RGB cameras or multispectral imaging systems, which compromise image spectral resolution in favor of much higher spatial resolution. This ultra-high spatial resolution is particularly relevant to detect very small weed seedlings in their earliest stages, which is generally the optimal time for implementing SSWM strategies. In these scenarios, ML algorithms tackled previously unsolved challenging tasks such as: 1) distinguishing weeds outside and inside crop rows with k-NN, SVM or k-means clustering in sunflower (Pérez-Ortiz et al., 2015) and in maize (Pérez-Ortiz et al., 2016), or with an ensemble of RF trees in sunflower and cotton (De Castro et al., 2018); 2) discriminating between broad-leaved and grass weeds in sunflower and cotton by using ANN-based MLP (Torres-Sánchez et al., 2021); 3) mapping bermudagrass patches in vineyards with cover crops by using DT (de Castro et al., 2020); and 4) spectral analysis and mapping of blackgrass weed in wheat parcels by using feature selection and RF with Bayesian optimization, respectively (Su et al., 2022). In the domains of crop diseases and plagues, relevant studies with UAV multispectral imagery are mainly focused on classifying crop/tree area damaged by a disease infestation or a plague attack, e.g., detecting bacterial fire blight *(Erwinia amylovora)* disease in apple or in pear trees with a combination of dimensionality reduction (mRMR), anomaly detection (isolation forest) and classification (DT, RF, SVM) algorithms (Xiao et al., 2022), or by using SVM classifier with RBF (Bagheri, 2020), respectively, discriminating bacterial (banana xanthomonas wilt - BXW) and viral (banana bunchy top virus - BBTV) diseases in banana plantations with the RetinaNet model based on the ResNet50 architecture as detector and the VGG16 architecture pre-trained with the ImageNet dataset as classifier (Selvaraj et al., 2020), and classifying cotton pixels affected by two-spotted spider mite attacks with the CNN-based AlexNet algorithm (Huang et al., 2018).

Advances in CNN algorithms have greatly promoted the use of field imaging systems and proximal sensing for precision crop protection applications in the last years, as a tool to improve classification accuracy in complex crop/pest scenarios (Barbedo, 2020) and to implement real-time applications (Rakhmatulin et al., 2021). In fact, recent innovations in agricultural robotics and weeding systems are based on CNN classifiers for pest detection and classification (Oberti and Schmilovitch, 2021; Allmendinger et al., 2022; Gerhards et al., 2022; Li et al., 2022). Some recent studies in the weed domain are the classification of *Chenopodium album* in potato fields by comparing CNN-based GoogLeNet, VGG-16 and EfficientNet (Hussain et al., 2021) and of five different weed species in tomato fields with CNN-based RetinaNet, Faster RCNN and YOLOv7 (López-Correa et al., 2022), in the disease domain are the early detection of fungal head blight *(Fusarium)* disease in wheat by applying CNN-based VGG and RNN classifiers to on-ground hyperspectral images (Jin et al., 2018) and diagnosing of fungal *black rot, black measles* (esca) and *leaf blight* diseases by applying CNN-based AlexNet, MobileNets and ShuffleNet to a repository of RGB images of grape leaves for potential use in mobile devices (Tang et al., 2020), while in the plague domain are the detection and classification of multi-class plague species in trap images by using CNN-based ZF, VGG16, ResNet50 and ResNet101 (Liu et al., 2019) and the detection of *Helicoverpa assulta, Spodoptera litura* and *Spodoptera exigua* moths in pheromone trap images by comparing Faster RCNN, R-FCN ResNet, Retinanet and SSD Inception classifiers (Hong et al., 2020), among many other case studies in the three crop protection domains.

## 5 Emerging technologies of precision crop protection in line with AG5.0

Crop protection has used technology to reinvent itself over time, with AI tools and ML algorithms being the main drivers in the last decade towards the implementation of automated, smart and precise tasks following the precision agriculture and digital Ag4.0 paradigms. While AI involves the scientific and technological research of machines that are able to perceive, reason, learn, adapt, make decisions and act rationally to meet objectives in a given environment, the advances in ML are behind the recent rise of AI in primary, industrial and service sectors. As discussed above, many of the ML algorithms have already been successfully applied in agriculture and other disciplines (**Table 2**), while others unprecedented in agriculture are now reaching the level of maturity needed to address new precision crop protection goals in line with emerging Ag5.0.

These goals will primarily focus on developing and exploiting two issues: 1) early detection of crop pests, and 2) autonomous real-time multitasking systems. On the one side, the former will enable the application of more effective control measurements at the optimal time before the damage provoked by a disease, weed or plague becomes too severe. The development of data-driven early detectors is particularly urgent considering the adverse effects that current climate change scenario are causing on cropping systems due to the spread of newly emerging or invasive pests (Juroszek et al., 2020; IPPC Secretariat, 2021). To this end, the implementation of Ag5.0 technologies will facilitate data fusion from various sources and tools (e.g. climate data, proximal and remote sensing, crop and soil sensors, farm management information systems, etc.) and assess the spatio-temporal occurrence and severity of the pests (Shankar et al., 2020), which will lead to improve early detectors and diagnostic algorithms (Picon et al., 2019; Ramcharan et al., 2019). On the other side, the latter aims the design of powerful autonomous systems capable of simultaneously doing the three main stages of precision crop protection in real time (see section 2), i.e. identifying occurrences of crop diseases, weeds or plagues at different spatial and temporal scales, analyzing crop and pest information, and make the decision of applying a customized site-specific management adjusted to each crop-pest scenario (Pretto et al., 2019; Birrell et al., 2020; Lottes et al., 2020).

**Figure 5:**
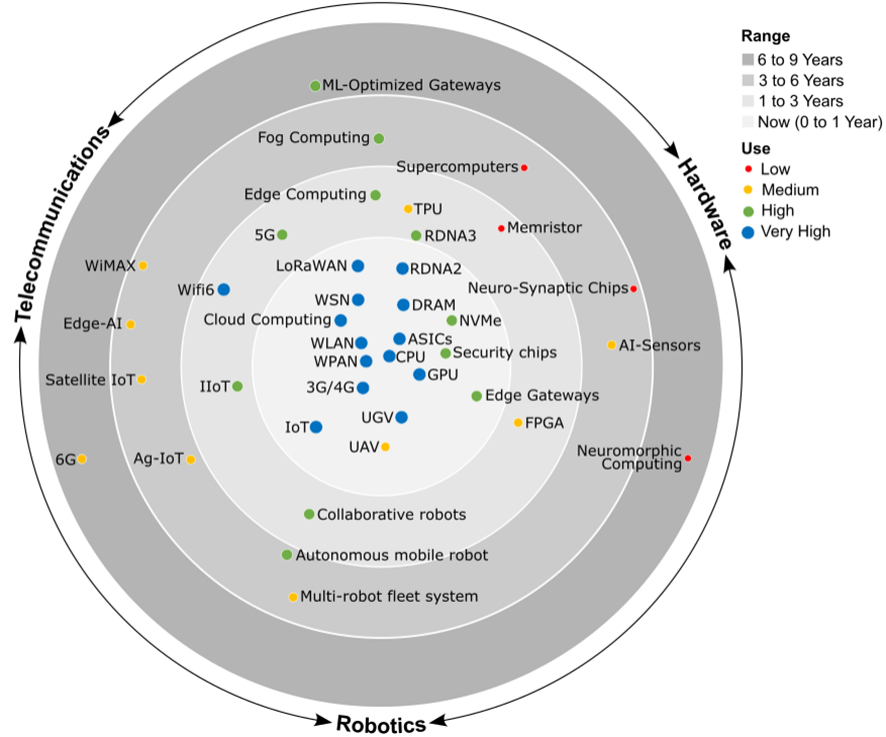
Multidisciplinary technological domain of Ag5.0 with a different degree of maturity and use ranging from mature technologies in the core circle to future technologies in the peripheral circle. CPU, central processing unit; GPU, graphics processing unit; TPU, tensor processing unit; DRAM, dynamic random-access memory; RDNA, radeon DNA; NVMe, non-volatile memory express; ASIC, application specific integrated circuit; FPGA, field programable gate array; LPWAN, low power wide area network; WLAN, wireless local area network; WPAN, wireless personal area network; WSN, wireless sensor network; IoT, internet of things; IIoT, industrial IoT; Ag-IoT, agricultural IoT; LiFi, light fidelity; WiMAX, worldwide interoperability for microwave access; TSN, time-sensitive networking; xG, cellular network generation.

Ag5.0 technology will tackle these and other future challenges with a multidisciplinary domain that relies on powerful ML algorithms (Liakos et al., 2018; Coulibaly et al., 2022), along with the latest technological solutions on hardware (Bustio-Martínez et al., 2022), telecommunications (Chopra et al., 2017; Ejaz et al., 2020), and robotics (Ren et al., 2020; Albiero et al., 2022), which may contribute now or in the short, medium or long term given their different degrees of maturity and use (**Figure 5**), as discussed below.

### 5.1 Hardware solutions for precision crop protection

Hardware tools are moving agriculture disciplines into digitization with innovative smart sensors, IoT ecosystems, architectures for specialized graphics processing, multicore embedded systems, and a number of new electronic devices, focused on the acquisition and use of crop data (Muhammad et al., 2019). The convergence of technologies is enabling to turn traditional agricultural sensors into smart sensors with built-in AI processing, that is, AI-Sensors with a dedicated chip embedded in the same sensor that can process ML tasks and, for example, may simultaneously perform object perception and analysis. Sony IMX500 and IMX501 (Sony Group Corporation, Tokyo, Japan) are two commercial image-based AI-sensors (Sony, 2020), in which the acquired signals are executed with a digital image signal processor at high-speed by the logic chip (i.e., 3.1 millisecond processing by the MobileNet V1). This processing speed is feasible as the sensor generates semantic information belonging to the image metadata instead of the image information, reducing data volume. In crop protection, these AI-sensors would facilitate the detection, recognition and control of targeting areas of crops with specific pest problems in real-time (e.g., weed species identification) and following optimized DSS prescriptions.

Advances in architectures for specialized graphics processing, such as GPU, TPU, radeon DNA (RDNA), in dynamic random-access memories (DRAM) and in communication and storage access protocols (e.g., non-volatile memory express, NVMe) are enabling greater programmability, opening up a wide range of Ag5.0 applications based on virtual modeling, the creation of digital twins and the use of supercomputers. A digital twin is a multi-physics, multi-scale, probabilistic simulation of a complex system that uses the best available physical models and sensor updates to reflect the life of its corresponding twin (Glaessgen and Stargel, 2012). While simulation-based analysis within a digital twin will lead to the development of innovative and more powerful DSS tools for precise pest management, the use of supercomputers will enable the study of crop-pest models in less time and drastically improve the performance of ML detectors and classifiers of crop diseases, weeds or plagues. Currently, the bottleneck to implement CNN-based architectures with high capacity for knowledge generalization is the training stage with large training datasets, but supercomputers will assist in overcoming this weakness by increasing the input data (data augmentation) and decreasing the computational time for model creation.

Performance of CNN-based architectures can be also improved with the use of Field Programmable Gate Array (FPGA), which enables the implementation of logic functions and is the basis for the creation of multicore embedded systems (Qiu et al., 2016; Shawahna et al., 2019). This technology will benefit precision crop protection with the development of new software applications running in operating systems used in agriculture IoT (Ag-IoT) solutions (Zhang et al., 2020) and the adaptation of customized pest detectors to mobile devices.

The devices connected to IoT systems are potentially risky in the absence of security elements or algorithms (Ibrahim and Gebali, 2022), reason why the devices performing edge gateway functions have improved designs with the use of application-specific integrated circuits (ASICs), just as security chips are essential in the implementation of Industrial IoT (IIoT) (Oñate and Sanz, 2023) and Ag-IoT systems. Wide evolution and adaptability of ML algorithms lead to their employ in optimizing gateway equipment tasks (ML-Optimized Gateways), making the performance of these tasks efficient even with resource limitations. The use of ML-Optimized Gateways in Ag5.0 will allow optimizing edge computing devices, reduce latency and increase privacy, which will result to create more efficient and safe models.

The future of both hardware and software solutions may reach a turning point in the medium term with the application of the computing principles derived from the memristors (Strukov et al., 2008). These devices are composed of two terminals with three layers, i.e. two electrodes for the communication of electrical signals and one storage layer that can be dynamically reconfigured when the inputs are stimulated, enabling data storage and direct processing (Zidan et al., 2018). The functioning of memristive elements is similar to that of neuronal synapses, becoming the technological basis of neuromorphic computing (Xia and Yang, 2019) and Spiking Neural Networks (SNNs) research (Jeong and Shi, 2019), which relies on a new neuron that is characterized by having a time-varying internal state, known as spiking neuron (Brette et al., 2007; Ghosh-Dastidar and Adeli, 2009). SNNs are the artificial representation that most closely emulate the brain, differing from ANNs in the incorporation of time as an explicit dependency in computations (Davies et al., 2018). Comparing to ANNs, SNNs achieve lower latency classifications, shorter computation times in the training phase, high accuracies and low energy consumption (Diehl et al., 2015; Esser et al., 2016), which can foresee that neuromorphic computing and SNNs will be the future tools to develop computational systems and create new electronic devices with a high impact on Ag5.0 technology.

### 5.2 Telecommunications for precision crop protection

Precision crop protection is increasingly heading towards a system-of-systems approach with multiple connected practices to achieve an integrated crop management strategy, in which on-ground, proximal and remote sensing are key technologies to assess and monitor all the biotic and abiotic factors that might affect crop health. In this framework, telecommunications are essential to connect devices (i.e., platforms, processors, actuators) and transfer data acquired by sensors, creating a networking environment that adds value in the tasks of data processing, pest prediction, decision-making, and crop management.

Wireless Sensor Networks (WSN) are leading communication systems in agriculture with various technologies that differ from each other mainly in their operating mode and specifications in terms of frequency range, transfer rate and power consumption (Thakur et al., 2019). Bluetooth and Zigbee (developed under IEEE 802.15.1 and 802.15.4 standards, respectively) are characterized by open specification, short range operation, high level data transmission with low latencies and low power consumption (Khanji et al., 2019; Zeadally et al., 2019). Zigbee covers larger distance (<100 m) than Bluetooth (<10 m), although data transfer is faster in Bluetooth (1-24 Mbps) than in Zigbee (40-240 Kbps). The alternatives to increase the range of operation and data transfer are the wireless fidelity (Wi-Fi) system, generally used for local area networks with a range of 50-100 m or even several hundred meters, and the worldwide inter-operability for microwave access (WiMAX) system used as a long-distance communication solution (up to 50 km). The development of IoT and the advance of low power wide area networks (LPWAN) are promoting the Long Range (LoRa) radio communication system and the LoRaWAN protocol as the most promising technology in agricultural disciplines (Castro et al., 2023), because of its long-range data transmission (dozens of kilometers, very useful in rural areas), low power consumption and secure connectivity (Gu et al., 2020). LoRaWAN uses a modified frequency modulation, operates in the Industrial, Scientific and Medical frequency band defined according to the geographical area (Asia 433 MHz, Europe 868 MHz and America 915 MHz), hence the sensors can operate in the license-free bandwidth (Lavric, 2019).

High-speed and efficient telecommunications are essential to implement real-time operations in actuator platforms (i.e., tractors, self-propelled sprayers, unmanned ground vehicles (UGVs), UAVs, etc.) that are focused to simultaneously percept, analysis and treat pest occurrences. In engineering and computer science, the concept of real-time is given to those processes whose execution, measured as the ratio between the input and output of a variable, occurs at very low time values (^<^milliseconds), therebeing a difference between real-time system and real-time computer system (Poniszewska-Maranda et al., 2020). Cloud computing, edge computing and edge AI are the three technologies to implement real-time actions on actuator platforms for precision crop protection in line with Ag5.0

Cloud computing is the convergence of information technology and business activity to provide services over the Internet. Companies such as Amazon, Google and Microsoft compete in the continuous improvement of infrastructures, hardware, computer security and high information processing (Mahmoud and Xia, 2019). To perform precision crop protection operations in real-time using cloud computing, the information collected with a sensorized platform must first be transmitted to the Internet, then processed and analyzed on any ML-based cloud service, and finally the prescription returned to the same platform to implement the actuation. These interactive operations need access times as short as possible, very close to real-time, to meet users’ demands, for which network architectures for wireless connections enabling Internet access such as 5G are already underway, with a view to the upcoming development of 6G. For example, the integration of 5G and future 6G with UAVs has enormous potential to apply precise aerial treatments of weed patches and eventually other pest occurrences following real-time detection (Ullah et al., 2020). Technical aspects aside, security and privacy issues are of particular concern in cloud computing systems, as infrastructures and applications may be subject to malicious attacks, as reported by Maniah et al. (2019) and Sun (2020). Indeed, privacy-sensitive reasons together with the progressive increase in data volume due to the connection of more devices has led to the introduction of fog computing, which allows decentralized processing, low latency and high bandwidth (Bonomi et al., 2012).

As mentioned before, current research is focused to platforms that detect, process and treat at the same time, which require a high computational cost in the limiting conditions of an equipment located on the farm, using the encoding method for signal transmission and taking into account the latency time of the radio transmission equipment. In this scenario, edge computing systems is a viable option as they allow the computing process to be performed close to the data source without the need for an Internet connection, thus avoiding data transmission problems and providing superior privacy and security, as well as reducing communication costs and energy consumption given the huge number of computations performed in ML modeling (García-Valls et al., 2018). A further step in the development of this computational architecture is offered by the devices for AI on the edge (Edge AI), which are embedded systems equipped with ML algorithms. Edge Intelligence is still at an early stage of research (Zhou et al., 2019), but is attracting great interest across all technological disciplines, with enormous potential in the development of agricultural robotics and autonomous crop protection treatments, since AI chips have achieved a high calculation capacity in the implementation of CNNs (Gao and Zhou, 2019).

### 5.3 Robotics for precision crop protection

Autonomous mobile robots (AMRs) allows the industry to increase productivity by doing more with fewer people, having great potential for boosting precision crop protection strategies in line with Ag5.0. AMRs have the ability to navigate with little or no human intervention under their control, in partially unknown environments (Alatise and Hancke, 2020). Therefore, their locomotion, perception, cognition and navigation systems must be able to address dynamic crops in position and time; in addition to: i) providing solutions to labor shortages, and ii) acquire real-time data for data-driven decision making, with the aim of significantly increasing yields within sustainable production (Shamshiri et al., 2018).

Several research projects have been developed to link robotic platforms to agricultural activities (Wolfert et al., 2017). In order to have completely robotized agricultural fields, robots must be able to adapt to the external environment and to the different types of land surface. Due to the great technological advances implemented in recent years, some robotic agricultural activities are already becoming commercially available (Lowenberg-DeBoer et al., 2020; Saiz-Rubio and Rovira-Más, 2020; Santos Valle and Kienzle, 2020; Sparrow and Howard, 2021; Botta et al., 2022), being the use of UGVs and UAVs that detect weeds and act in real-time with high precision the most popular robotic system to implement a precision crop protection activity (Oberti and Schmilovitch, 2021; Li et al., 2022). Some AMRs with great potential are: 1) RIPPA (Australian Centre for Field Robotics, The University of Sydney, Austria), based on the design of their previous robot LADYBIRD, uses an intelligent perception system and is equipped with a variable injection precision applicator, with an operating autonomy of twenty one continuous hours (Bogue, 2016); 2) AgBot-II (Queensland University of Technology, Brisbane, Australia) with a vision system not only detecting but also classifying weed species in real time, then using the Inception-v3 architecture as its DDS, which allows to decide the weed management method to apply, either mechanical, chemical or a combination of both, weeds on accuracies over 90% (McCool et al., 2018); 3) Robotti (Agrointelli, Aarhus, Denmark), whose module-based construction allows it to operate in various soil environments, adapting to different types of crops (Grimstad and From, 2017); 4) AVO (Ecorobotix, Yverdon, Switzerland) that uses CNNs algorithms for the detection and selective control of weeds by herbicide spraying in real time, obtaining a detection rate of 85% (https://ecorobotix.com/en/avo/); 5) BONIROB (AMAZONE Technology Leeden GmbH & Co. KG, Germany) (https://info.amazone.de/DisplayInfo.aspx?id=29417) with an integrated system using camera-based machine vision, image processing to detect the plants and a sprayer with individually controlled valves, allows selective and precise control of weeds, thus achieving both ecological and economic advantages; 6) Kilter AX1 (Kilter AS, Norway) (https://www.kiltersystems.com/ax1) uses machine vision combined with AI and a novel nozzle technology that applies a micro-drop (6×6mm resolution), which allows to reduce the amount of herbicides up to 95%; 7) DINO (Naïo Technologies, France) (https://www.naio-technologies.com/en/dino/), a weeding robot with an accuracy of 2 cm achieved by the RTK GPS system that has a vision system to detect the crop rows and adjust the position of the mechanical weeding tools in row, allowing high precision weeding and hoeing; 8) Odd.bot (Odd.Bot B.V., The Netherlands) (https://www.odd.bot/), a mechanical in-row weeding robot that relies on machine vision and AI-based seedling recognition; 9) Titan FT35 (FarmWise Labs Inc., USA) (https://farmwise.io/) uses machine vision and ML algorithms trained to learn the characteristics of crops such as broccoli, lettuce, cauliflower and tomatoes to differentiate between the crop and weeds; it has six internal weeders with blades that eliminate weeds with centimeter accuracy; and 10) FARMING GT (Farming revolution GmbH, Germany) (https://farming-revolution.com/) distinguishes weed seedlings with 99% reliability in different crops (e.g., cabbage, lettuce varieties, onions, corn, sugar beet, pumpkin, field bean, potato, canola, soybean, wheat), then carrying out in-row and inter-row mechanical weeding.

Collaborative or cooperative robots will support the future development of Ag5.0 (Lytridis et al., 2021). These robots are designed to complement the routine activities by improving their ergonomics (Pauline et al., 2019) and also sharing the workspace. An advanced application of collaborative robots is in organic food production, particularly in pest control with nonchemical methods by using robotic mechanical control (Machleb et al., 2020) and viable handling systems for harvesting (De-An et al., 2011; Zhang et al., 2021), which has been shown as a solution to increase the benefits of organic crop management (Pérez-Ruíz et al., 2014; Giampieri et al., 2022). The development of Ag5.0 will allow the convergence of UGV and UAV systems, for their collaborative and cooperative operation under a unified control, giving rise to Multi-robot Fleet Systems (MFS). Workload performed by several small robots composing a MFS is equivalent to that developed by a larger machine, highlighting that the MFS have a more precise positioning (de Santos et al., 2017).

Current technology has allowed the development and maturation of sensory-motor autonomy, reactive autonomy and cognitive autonomy in UAVs (Floreano and Wood, 2015), making them a great tool that together with RGB, multispectral, and hyperspectral sensors facilitate the acquisition of information on plant diseases, weeds, and plagues. That is why in Ag5. 0, detection and actuation systems based on ML algorithms and implemented in embedded systems will be part of the UAVs. ML techniques within Ag5.0 will allow the integral management of fleets of autonomous vehicles (UAV and UGV) decentralized in real time, besides being the basis for the implementation of robust navigation systems, such as the redundant system developed by (Belhajem et al., 2016) where they used ANNs in conjunction with genetic algorithms and the Extended Kalman Filter to reliably estimate the position of a vehicle in real time in the absence of GPS signal. The objective of having fleets of autonomous vehicles is the application of specific treatments for the detection and action on weeds and others pests (Emmi et al., 2014), which will finally reduce production costs and reduce the environmental impact of the use of herbicides and pesticides.

## 6 Conclusions

This article provides a framework on the future direction of precision crop protection, with a focus to scientific, agronomic and industrial applications of traditional ML algorithms and recent advances in the ANNs models. In the period 2010-2022, 125 algorithms applied in all disciplines were identified, of which 122 were used in the domains of crop diseases, weeds and plagues, with the aims of solving tasks on classification, regression, clustering, anomaly detection, dimensionality reduction, and association rule learning, and moving precision crop protection closer to the emerging concept of Ag5.0. This process should be accompanied by innovations and dedicated solutions in the areas of hardware, telecommunications and robotics, some of which are already being implemented in agriculture and others are still unprecedented, as this article outlines by introducing 39 emerging technologies and citing some 80 scientific and technical references. The transition from current Ag4.0 to future Ag5.0 strategies in the field of precision crop protection will be driven mainly by their focus and level of automation. Ag5.0 will promote a new era of intelligent crop management with a greater emphasis on solving complex crop protection objectives (e.g. early detection of crop pests) and enhancing management practices (e.g. autonomous real-time multitasking) as a whole, with a main focus to automatized decision-making processes, unmanned operations and progressively less human intervention supported by the latest AI systems, advanced robotics, and powerful ML algorithms.

## Author contributions

MP-O, JD, and JP conceived and designed the review; GM-R conducted the bibliographic search with support of JD, AC, and JP; GM-R, and MP-O analyzed the data and defined the ML taxonomy; JD, AC, and JP selected the case studies cited; GM-R and JP identified the emerging technologies; GM-R, JD, and JP wrote the first draft. All authors contributed to the article and approved the submitted version.
